# Home Food Safety Practice and Household Food Insecurity: A Structural Equation Modeling Approach

**Published:** 2019-10

**Authors:** Fatemeh ESFARJANI, Hedayat HOSSEINI, Ramin KHAKSAR, Roshanak ROUSTAEE, Haleh ALIKHANIAN, Marjan KHALAFI, Amin MOUSAVI KHANEGHAH, Fatemeh MOHAMMADI-NASRABADI

**Affiliations:** 1. Department of Food and Nutrition Policy and Planning Research, National Nutrition and Food Technology Research Institute, Faculty of Nutrition Sciences and Food Technology, Shahid Beheshti University of Medical Sciences, Tehran, Iran; 2. Department of Food Science and Technology, National Nutrition & Food Technology Research Institute, Faculty of Nutrition Sciences and Food Technology, Shahid Beheshti University of Medical Sciences, Tehran, Iran; 3. Department of Food Sciences, Faculty of Food Engineering, University of Campinas (UNICAMP), Campinas, Sao Paulo, Brazil

**Keywords:** Household food insecurity, Home food safety practice, Structural equation modeling

## Abstract

**Background:**

Food safety and food security are interrelated concepts with a profound impact on the quality of human life. The current study, for the first time, was set to identify associations between home food safety practice and household food insecurity a structural equation modeling approach.

**Methods:**

In this cross-sectional study, urban households were selected from among 10 health centers of five districts of Tehran, Iran (2015). The following questionnaires were completed: socioeconomic status (SES), food security and food safety. Structural Equation Modeling (SEM) was applied for predicting the relationships between SES, food insecurity, and food safety in households*.*

**Results:**

Food security was observed in 56% of households. Mild, moderate and severe food-insecure households were determined to be 29%, 12%, and 3%, respectively. In addition, the scores of home food safety practice in 37.5%, 33% and 29.5% of the households were classified as desirable, acceptable and weak, respectively. Low-educated mothers having husbands with low educational and occupational level had a weaker food safety practice compared to high-educated ones. Based on the SEM results, an inverse association between food safety practice and food insecurity score was observed (t= −2.89, ɣ = 0.16).

**Conclusion:**

Food insecurity and undesirable food safety practice were relatively prevalent among households. In addition, the economic and social factors could inversely affect both food insecurity and food safety practices.

## Introduction

Food safety is not only related to safe food but also deals with safe consumption of food products (1). According to a meta-analysis study conducted in Iran, the rate of mild, moderate and severe FI in this country reported being 28.6%, 14.9% and 6%, respectively (2).

In 2014, 2797 cases of foodborne diseases have been identified in Iran from which 63 cases resulted in death based on an unpublished report of the Ministry of Health and Medical Education (3). Implementing food safety is relatively difficult, which would be an even greater challenge when compounded with food insecurity (4).

Many external factors affect food safety and food security, interrelated concepts, and both of them have affected the quality of human life. Unsafe food items can cause several illnesses, so food safety, nutrition and food security are well situated among the 13 defined strategic goals by the WHO (5). One of the major problems of home food safety in Iran is lack of knowledge regarding food handling, storage and hygienic practices, which may lead to food-borne illnesses (6). There are limited studies that provide insights into the association between the status of food security and home food safety among households (7–10). The objectives of this study were investigation of the relationship between food safety and food security, and evolution of the impact of some factors such as socioeconomic status (SES).

## Materials and Methods

### Study design and subjects

This cross-sectional study was conducted among the households of Tehran, Iran (2015). Districts were chosen based on the socioeconomic status of the residents. Totally, 655 households were selected by simple random sampling from 10 health centers in five districts of Tehran. Twenty-five out of 655 households were eliminated for not willing to participate in the study. Ultimately, 630 women consented to enroll in the study.

The participants' inclusion criteria were defined as women, registered, frequently received health care services from the health centers, and were responsible for food handling in their households. According to the timetable schedule, the health centers' staffs contacted the households by phone to invite them there, and well introduced the purpose of the study.

### Ethical aspects

Informed consent was obtained from all participants, and they were informed that their responses would remain anonymous and confidential. Additionally, they were informed that participation in the study was voluntary and they had the right to withdraw from the study at any time.

### Questionnaire

Tri-Sectional questionnaires were completed via face-to-face interviews by trained interviewers in health centers. As the first part of the questionnaire, SES, defined by composite indicators including household appliances (housing material, car, motorcycle, computer, color television, freezer, vacuum cleaner, washing machine, and furniture), average monthly expenditure (per capita), age, educational and occupational level, family size, household income, residential infrastructure, and residency living conditions.

The Home Food Safety Practice Questionnaire (HFSQ) including personal hygiene, food safety, preparation storage and safety of cooked food (11-items) was applied as the second part of the questionnaire. The validated questionnaire was derived from the first phase of the present study entitled “Determinants and predictive modeling of home food safety practices households of Tehran” using mixed methods approach (11). The scores were categorized into three levels according to the following three tertiles: desirable [51–55], acceptable [47–50], and weak [<46].

Finally, as the third part of the questionnaire, the validated Household Food Insecurity Access Scale (HFIAS) was used (12). HFIAS includes a 9–item questionnaire, which asks whether a specific condition associated with the experience of food insecurity (FI) has ever occurred during the past 30 d. Households were grouped into four categories based on their scores: secure [0–1], mildly [2–7], moderately [8–14], and severely food insecure [15–27].

### Data analysis

After data entry, a check was made for any errors, including coding numbers, typographical errors, then statistical analysis was performed by the SPSS software (ver. 16, Chicago, IL, USA). Descriptive data were presented as frequencies and mean (±SD), tested by Chi-square and analysis of variance (ANOVA). The explored causal relationships between SES, FI, and food safety in the households were justified using Structural Equation Modeling (SEM) conducted by LISREL 8.5 software. The Goodness of fit indices of the proposed model and the path coefficients were estimated using maximum likelihood. The “t” values greater than 2 were considered as significant. The X2/df ratio of 2.00 or less, Goodness of fit indices (GFI, AGFI) higher than 0.95, and PMSR near to 0.05 were considered as a Good fit (13).

## Results

Demographic and socioeconomic characteristics of the studied households show in Table 1. Low-educated mothers having husbands with low educational and occupational level had a weaker food safety practice compared to high-educated ones. Households which living in the central parts of Tehran had more desirable food safety practice. Moreover, mothers from food insecure households had weaker food safety practice than food secure ones.

**Table 1:** Qualitative socio-economic characteristics of studied households based on food safety practice in Tehran

***Demographic variables (n= 630)***	***Food Safety practicen (%)***			***Total n (%)***	P-value*
	Weak (<46)	Acceptable (47–50)	Desirable (51–55)		
Mother's education					0.000
Illiterate/ Primary	74(50.7)	45(30.8)	17(18.5)	146(100)	
Secondary school to high school	83(24.3)	119(34.9)	139(40.8)	341(100)	
University	29(20.3)	44(30.8)	70(49)	143(100)	
Mother's job					0. 943
Housewife	171( 29.6)	191(33.1)	215(37.3)	577(100)	
Employer	15(28.3)	17(32.1)	21(39.6)	53 (100)	
Father's education					0.000
Illiterate/ Primary	50(43.1)	35 (30.2)	31 (26.7)	116(100)	
Secondary school to diploma	96(27.5)	112(32.1)	141(40.4)	349(100)	
University	30(22.7)	48(36.4)	54(40.9)	132(100)	
Father's job					0.003
unemployed	5(31.3)	3(18.8)	8(50.0)	16 (100)	
Retired	15(26.3)	23(40.4)	19(33.3)	57 (100)	
laborer	33(32.7)	41(40.6)	27(26.7)	101(100)	
Freelancer	82(32.9)	85(34.1)	82(32.9)	249(100)	
Employee	32(22.1)	37(25.5)	76(52.4)	145(100)	
Manager	9(31.0)	6(20.7)	14(48.3)	29 (100)	
District					0.006
North	24(19.8)	51(42.1)	46(38.0)	121(100)	
Center	31(24.6)	38(30.2)	57(45.2)	126(100)	
East	60(39.2)	44(28.8)	49(32.0)	153(100)	
West	46(34.1)	37(27.4)	52(38.5)	135(100)	
South	25(26.3)	38(40.0)	32(33.7)	95(100)	
Food security households					0.003
Secure (0–1)	83(23.6)	120 (34.2)	148 (42.2)	351(100)	
Mild (2–7)	62(33.7)	60 (32.6)	62 (33.7)	184(100)	
Moderate (8–14)	34 (44.2)	25( 32.5)	18( 23.4)	77 (100)	
Severe (15–27)	7 (38.9)	3 (16.7)	8 ( 44.4)	18 (100)	
Total	186(29.5)	208(33)	236(37.5)	630(100)	

* Chi-square test

Table 2 shows the quantitative of socio-economic characteristics of studied households based on their food safety practice.

**Table 2:** Quantitative socio-economic characteristics of studied households based on food safety practice in Tehran

***Demographic variables (n= 630)***	***Food safety practice mean (SD)***			***Total Mean (SD)***	P-value*
	Weak (<46)	Acceptable (47–50)	Desirable (51–55)		
Mother's age (yrs)	37.0(12.7)	35.6(11.5)	36.0(10.4)	36.16(11.5)	0.478
Family size	3.4(1.0)	3.5(1.0)	3.4(0.87)	3.0(0.97)	0.364
Income ^†^	675(350)	687(325)	725(350)	700(350)	0.225
Expenses ^†^	246(146)	256(162)	265(164)	256(158)	0.451
Food expenses^†^	127(75)	141(88)	136(84)	135(83)	0.253
Home appliances^‡^	2.9(1.6)	3.1(1.6)	3.1(1.7)	3.1(1.7)	0.220
Floor area (m^2^)	75.1(43)	74.0(36)	75.0(31)	74.7(36)	0.942

* ANOVA test //

† US $ per month for each household //

‡ Including freezer, microwave, dishwasher, car, motorcycle, computer/laptop, and Internet access

There are no significant differences (*P*>0.05) between food safety practices and all socio-demographic variables tested. Prevalence of secure, mild, moderate, and severe food-insecure households was 56%, 29%, 12%, and 3%, respectively (data was not presented).

**Fig. 1:**
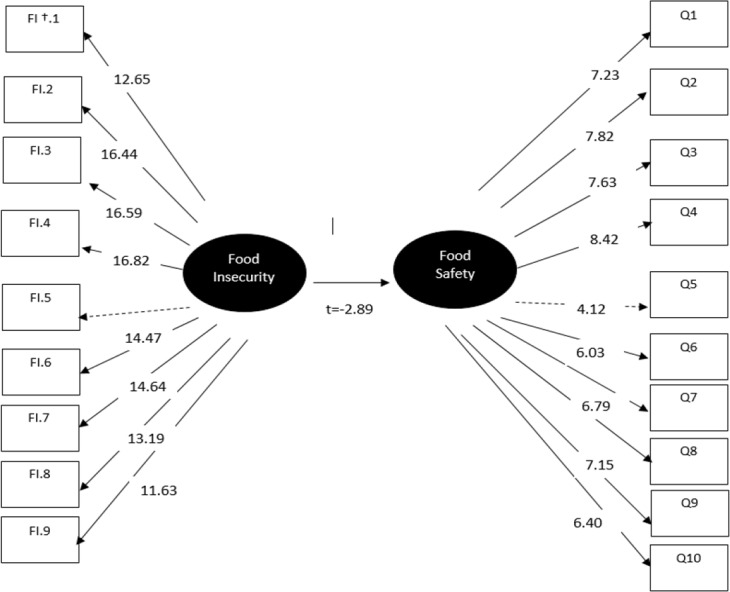
Structural equation modeling (SEM) of the relationship between food insecurity and food safety practice FI ^†^ = Food Insecurity **-** Q11 was removed because it was not significant in the model. // Chi-square=1211.07, df=169, *P*-value=0.00000, RMSEA=0.099 // 90% Confidence Interval for RMSEA = (0.091; 0.10) // Adjusted Goodness of Fit Index (AGFI) =0.81

**Fig. 2:**
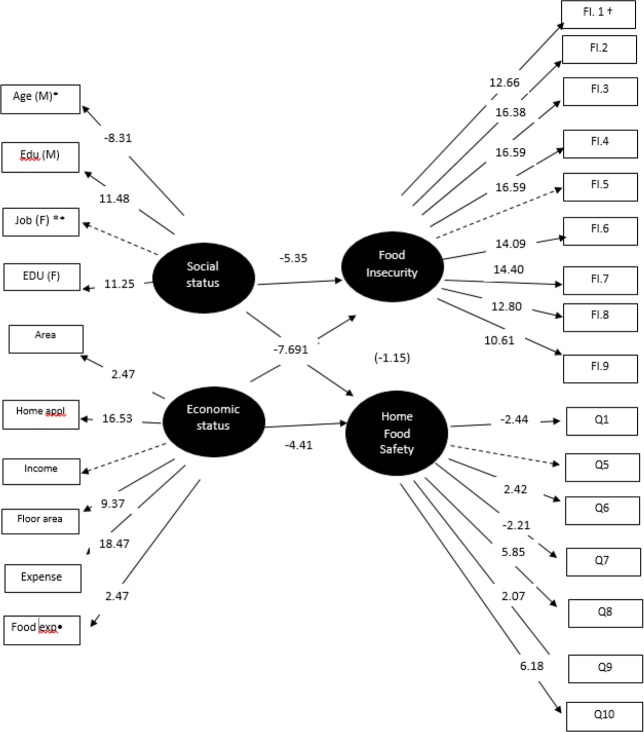
Structural equation modeling (SEM) of the relationship between food insecurity, food safety practice and social and economic factors in the examined households in Tehran *M=Mother, F**=Father, exp•= Food expenditure, FI ^†^= Food Insecurity **-**Q2, Q3, Q4 and Q11 were removed because there were not significant in the model. Chi-square=2672.28, df =399, *P*-value=0.00000, RMSEA=0.095 90% Confidence Interval for RMSEA = (0.091; 0.10) Adjusted Goodness of Fit Index (AGFI) = 0.81 Comparative Fit Index (CFI) = 0.88

As shown in Fig. 1, before including the social and economic variables in the model, a significant inverse relationship between food insecurity and food safety practice was shown (t= −2.89, ɣ = 0.16), whereas after including them in the model, social (age, education and job status) and economical (income, expenses, food expenditure, home appliance, and floor area) status remained significant, showing an inverse relationship between the socio-economic factors and both food insecurity and food safety practice; however, as the *“t”* values greater than 2 were considered significant, the relationship between food safety and food security was removed (t= −1.15, ɣ= −0.09) (Fig. 2).

## Discussion

Food safety is receiving heightened consideration worldwide as one of the essential links between food and health (14). Demographic and socioeconomic characteristics are important determinants for both food safety and food insecurity. The findings of the present study showed that the majority of young women, which their educational level was below diploma, have undesirable food safety practices. Increasing age could have positive effects on food safety practice (10). Low educational level and low income could lead to weak food safety practices (15, 16). In a meta-analysis, individuals with lower educational level were also negligent about food safety practices (17).

A few studies in Iran (18) and other countries (19–22) showed that the majority of women had undesirable food safety practices. Our findings further revealed that, during the last decade, home food safety practice has significantly improved in Iran (23,24). This could be attributed to the efforts from the international platforms such as WHO (for instance, food safety initiatives under the slogan “From farm to plate, make food safe” and other food safety campaigns in Iran) aiming to prevent food-borne disease (25). Based on the present study results, 44% of the households suffered from FI. In Thailand, 39% and 30% of the households experienced moderate and sever FI, respectively (26). In Malaysia, 66% of the households with low socioeconomic status experienced degrees of FI (27). The prevalence of FI among the Indian households with and without children was reported to be 57% and 43%, respectively (28). In Iran, 79% of the households in one of the counties of Iran were food insecure (29).

FI was 49.2%, implying that about half of Iranian households were food insecure (30). In Iran 17.5%, 14.4% and 11.8%, of the studied households were mild, moderately, and severely food insecure, respectively (12). Comparing with finding of the present study after 5 years showed an increase to 29% in the mild, a reduction to 12% in the moderate, and a decrease to 3% in the severe FI. Since Iran is currently undergoing a nutrition transition, leading to considerable variations in nutritional status within the population, consumption of low nutrient density foods has increased while dietary energy has increased, which could be due to the cash transfer program that can be one of the reasons for reduction the number of insecure households (31).

The inverse relationship between FI and average household income (32), maternal education (33) and unemployment (34) have been demonstrated previously (35, 36). In the present study, social and economic factors were reversely associated with household FI in the model. In Malaysia, the level of father's educational level was related to the household's FI, whereas there was no such association between the mother's educational level, job status and the household's FI (37). The combination of working experience (socialization with other people) and ability to generate and control financial resources in the households may allow the women to provide enough food for family members regardless of their education.

There are limited number of studies using the SEM in food safety and food security. The current study is one of the first investigations to explore the association between FI and home food safety practice using the SEM. A study using SEM in Malaysia indicated that food safety knowledge was negatively affected by food safety behavior, while food safety attitude firmly influenced food safety behavior in positive way (38). A study in Nigeria assessed factors affecting rural households' resilience to FI using the SEM approach. The indicators of asset and social safety nets had positive and significant impacts on the households' resilience to FI (39). A study entitled “measurement and modeling of household food security in Tehran using SEM” showed that before including the expenditure variable in the model, the most essential determinants of FI status of the households were house conveniences and housing conditions. However, after including the expenditures in the model, FI was reversely correlated with expenditures, and fruit and meat consumption (40).

Using SEM in the current study showed an inverse relationship between food insecurity and food safety practice. The economic and social factors could inversely affect both food insecurity and food safety practices. Therefore, special attention should be paid to the factors involved in promoting the health of the community in order to achieve sustainable development (41).

There were a few limitations to the study. The sample size was limited to the households living in the capital city of Tehran, which might not be generalizable to all the Iranians from various ethnic and socio-cultural backgrounds. Besides, there may be also under-reporting from a few participants who may not have reported their current household incomes and household food security accurately. Lack of data about calorie consumption and food intake can be considered as another limitation to this study.

## Conclusion

Undesirable food practices and FI were relatively prevalent among the households of Tehran city, and that the social and economic factors could inversely affect both food insecurity and food safety practices using the SEM. Policymakers should provide food safety education programs for food insecure households in order to reduce the possible risk of foodborne diseases. By establishing a national government plan, we can promote food security and food safety and thus give support to sustainable development.

## Ethical considerations

Ethical issues (Including plagiarism, informed consent, misconduct, data fabrication and/or falsification, double publication and/or submission, redundancy, etc.) have been completely observed by the authors.
